# Ministernotomy Versus Standard Sternotomy for Aortic Valve Replacement

**DOI:** 10.7759/cureus.76652

**Published:** 2024-12-30

**Authors:** Selman Dumani, Ermal Likaj, Laureta Dibra, Saimir Kuci, Edlira Rruci, Alfred Ibrahimi, Elizana Zaimi (Petrela), Alessia Mehmeti, Vera Beca, Devis Pellumbi, Aferdita Veseli, Ali Refatllari, Altin Veshti

**Affiliations:** 1 Division of Cardiac Surgery, University Hospital Center "Mother Theresa", Tirana, ALB; 2 Department of Anesthesiology, University Hospital Center "Mother Theresa", Tirana, ALB; 3 Department of Public Health, Faculty of Medicine, University of Medicine, Tirana, ALB; 4 Division of Nosocomial Infections, University Hospital Obstetrics and Gynecology "Queen Geraldina", Tirana, ALB

**Keywords:** aortic valve replacement, aortic valve surgery, minimally invasive, ministernotomy, postoperative recovery times

## Abstract

Background: Minimally invasive aortic valve surgery is becoming more popular everyday. The most used approach is mini-sternotomy. There are several promoted benefits related with minimal invasive approaches in comparison with standard sternotomy. This study aimed to compare the early postoperative results of mini-sternotomy and standard sternotomy.

Materials and methods: This is a retrospective study that compares minimal invasive with conventional sternotomy aortic valve surgery in terms of early results. The patients underwent surgery at the University Hospital Center “Mother Theresa”, Tirana, Albania between July 17, 2017, and July 30, 2024. The data were collected from hospital registration. All data are presented as mean ± standard deviation. Key outcomes included early mortality, perioperative complications, and intraoperative and postoperative recovery times.

Results: The study included 168 patients (95 males, 73 females) with a mean age of 62 ± 12.5 years. Standard sternotomy was used in 115 patients with a mean age of 63.93± 9.52 and mini-sternotomy was used in 53 patients with a mean age of 62.97 ± 10.47 without differences between them (P=0.633). The overall mortality was 1.2 % (four patients). There were no significant differences in mortality and incidence of perioperative complications between the two groups. The minimally invasive group had shorter intensive care unit (ICU) stay (39.92 ± 8.62 hours vs. 55.96 ± 32.56 hours, p < 0.001) and mechanical ventilation assistance duration (5.82 ± 2.44 hours vs. 10.41 ± 14.68 hours, p = 0.030).

Conclusions: The minimally invasive aortic valve replacement through mini-sternotomy is as safe as standard sternotomy. Mini-sternotomy clearly is related to significant shorter ICU stay time and mechanical respiratory assistance that can be traduced in lower hospital cost.

## Introduction

Aortic valve surgery is the most common procedure in heart valve surgery. While standard sternotomy remains the primary approach for the surgical treatment of aortic valve pathologies, minimally invasive valve surgery has emerged as a viable alternative. A minimally invasive approach is associated with several benefits for patients undergoing aortic valve surgery, including less trauma, less bleeding, shorter postoperative recovery, less pain, and better cosmetic outcomes. These benefits have been promoted since the inception of this approach in the mid-1990s [1-5]. However, skeptics argue that working in a smaller space may negatively affect the safety of a well-stabilized standard approach. This study aims to compare ministernotomy and standard sternotomy in terms of early postoperative results and the primary objective was to report the safety and benefits of this minimally invasive approach.

This work was presented as an oral speech, "Mini AVR versus full sternotomy AVR", at the Euro-Asian Bridge Meeting 2024, Iasi, Romania.

## Materials and methods

This was a retrospective study conducted at the University Hospital Center “Mother Theresa”, Tirana, Albania, and included patients who underwent isolated aortic valve replacement (AVR) between July 17, 2017, and July 30, 2024. This research article was approved by the Ethics Council, University of Medicine, Tirana (approval number: 3265/Prot, dated December 11, 2024).

Participants and data collection

All included patients were adults. Patients requiring surgical procedures other than an aortic valve were excluded. Additionally, all surgeries were performed by the same first surgeon. Patients operated by other surgeons as the primary surgeon were excluded. There was a total of 168 patients, who were divided into two groups: Group 1 included 115 patients who underwent surgery via standard sternotomy and Group 2 included 53 patients who underwent aortic valve surgery through ministernotomy. Demographic characteristics, key echocardiographic data, clinical status at admission, and operative and postoperative outcomes were gathered from hospital records.

Surgical techniques

The interventions, whether standard or ministernotomy, were conducted under general anesthesia. A standard sternotomy was performed using a conventional median sternotomy incision, followed by cannulation of the ascending aorta, and right atrium after full heparinization. After the aortic cross-clamping, a transverse aortotomy was performed, and the aortic valve was effectively exposed, removed, and replaced with an aortic valve prosthesis.

The minimally invasive approach utilized either a “J” right upper or an inverted “T” mini-sternotomy. The incision was approximately 7-8 cm long and extended from the sternomanubrial angle to the third or fourth intercostal space. Direct aortic and right atrial cannulation was achieved successfully. Cardioplegia was administered antegrade, and an oblique aortotomy was used to expose the aortic valve. Warm Calafiore blood cardioplegia was administered in both groups. The remaining procedural steps were the same as in standard sternotomy. In the ministernotomy group, we inserted the drainage tubes and epicardial pacemaker before weaning from the cardiopulmonary bypass. 

Data analysis

Statistical analysis was performed using IBM SPSS Statistics for Windows, Version 26.0 (Released 2019; IBM Corp., Armonk, New York, United States). The mean and standard deviation were used to analyze continuous variables. The Student’s t-test, Chi-squared, and Mann-Whitney test were used for statistical analysis. A p-value of less than 0.05 was deemed statistically significant.

## Results

Table 1 summarizes the general demographic data (age, gender, body surface area), clinical data at admission according to the New York Heart Association classification (NYHA), and preoperative main echocardiographic data (ejection fraction, pulmonary systolic artery pressure, calcification of aortic valve annulus) of the patients included in the study. The average age of the cohort was 62 ± 12.5 years, with no notable differences between groups (P=0.633) and approximately equal distribution of male and female patients. Observed pathologies included aortic valve stenosis (AVS), aortic valve regurgitation (AVR), and a combination of both conditions. The predominant pathology was AVS, with no significant statistical differences between the groups. A higher percentage of minimally invasive surgeries were urgent than the standard surgery group, indicating a significant difference (p = 0.009). A significantly higher proportion of patients in NYHA III were in the minimally invasive group of patients at admission (p < 0.001). Heavily calcified annuli were also significantly more prevalent in the minimally invasive group than in the standard surgery group (p = 0.008).

**Table 1:** General and preoperative data ‡: t-test; †: Mann Whitney u-test; *: Chi-square test; p˂ 0.05 statistically significant NYHA: New York Heart Association; PAsP: pulmonary artery systolic pressure; AVS: aortic valve stenosis; AVR: aortic valve regurgitation

General and Preoperative Data	Standard Surgery (n=115)	Mini-sternotomy (n=53)	Test value	p-value
Male, n (%)	68 (59.1)	27 (54.0)	0.376	0.540*
Female, n (%)	47 (40.9)	26 (46.0)		
Age (years), mean ± SD	63.93± 9.52	62.97 ± 10.47	2866	0.633†
Body Surface Area (m^2^), mean ± SD	1.84 ± 0.16	1.83 ± 0.16	0.268	0.789‡
Urgent, n (%)	15 (13.0)	15 (30.0)	6.736	0.009*
Elected, n (%)	100 (87.0)	35 (70.0)		
NYHA Class I, n (%)	3 (2.7)	0 (0.0)	21.016	<0.001
NYHA Class II, n (%)	69 (61.6)	13 (26.0)		
NYHA Class III, n (%)	38 (33.9)	34 (68.0)		
NYHA Class IV, n (%)	2 (1.8)	3 (6.0)		
Ejection Fraction (%), mean ± SD	59.37±10.35	61.66± 6.22	1.670	0.097‡
PAsP (mmHg), mean ± SD	40.66 ±14.62	35.40 ± 9.99	1.970	0.052‡
Aortic Valve Annulus (mm), mean ± SD	21.04 ± 1.68	21.22± 1.36	0.665	0.507*
AVS, n (%)	84 (73)	40 (75)		NS*
AVR, n (%)	6 (5)	2 (4)		NS*
AVS+AVR, n (%)	25 (22)	11 (21)		NS*
Heavily Calcified annulus, n (%)	61 (74.4)	43 (93.5)	7.048	0.008*

Table 2 compares comorbidities and risk factors between the two groups. The most common comorbidity in both groups was diabetes mellitus, with no significant differences observed.

**Table 2:** Risk factors and comorbidities *: Chi-square test, p < 0.05 statistically significant COPD: chronic obstructive pulmonary disease

Risk Factors and Comorbidities	Standard Surgery (n=115), n (%)	Mini-sternotomy (n=53), n (%)	Test value	p-value*
Hypertension	103 (90.4%)	44 (88.0%)	0.207	0.649
Renal Insufficiency	5 (4.4%)	1 (2.0%)	0.561	0.454
Diabetes Mellitus	24 (21.1%)	5 (10.0%)	2.917	0.088
Peripheral Artery Disease	2 (1.8%)	0 (0.0%)	0.888	0.346
Smoking	13 (11.4%)	7 (14.0%)	0.219	0.640
Obesity	3 (2.6%)	4 (8.0%)	2.451	0.117
COPD	6 (5.3%)	3 (6.0%)	0.036	0.849
Carotid Artery Disease	5 (4.4%)	1 (2.0%)	0.561	0.454

Table 3 presents the intraoperative times and postoperative times and complications. The cardiopulmonary bypass and cross-clamp durations were longer in the ministernotomy group, but only the CPB time reached a significant difference (P=0.002). The minimally invasive procedures had shorter ICU stays (39.92 ± 8.62 hours vs. 55.96 ± 32.56 hours, p < 0.001), and mechanical respirator assistance duration (5.82 ± 2.44 hours vs. 10.41 ± 14.68 hours, p = 0.030)). Atrial fibrillation was more prevalent in the standard surgery group, demonstrating a trend toward significance (p = 0.061). Overall mortality rates were low and comparable between the two groups, with the standard surgery group reporting two cases (1.7%) and the minimally invasive group reporting two cases (3.7%).

**Table 3:** Operative and postoperative results ‡: t-test; †: Mann_Whitney U test*: Chi-square test; p < 0.05 statistically significant CPB: cardiopulmonary bypass; XT: cross-clamping time; MRA: mechanical respiratory assistance; ICU: intensive care unit

Operative and Postoperative Results	Standard Surgery (n=115)	Mini-sternotomy (n=53)	Test value	p-value*
Mortality, n (%)	2 (1.7)	2 (3.7)	0.372	0.542
Low Cardiac Output, n (%)	2 (1.7)	0 (0.0)	0.931	0.348
Atrial Fibrillation, n (%)	16 (13.9)	2 (4.0)	3.524	0.061
Wound Infection, n (%)	2 (1.7)	2 (4.0)	0.753	0.386
Conduction Disorders, n (%)	6 (5.2)	0 (0.0)	2.707	0.100
Stroke, n (%)	1 (0.9)	0 (0.0)	0.437	0.508
Renal Insufficiency, n (%)	2 (1.7)	0 (0.0)	0.880	0.348
Hemorrhage, n (%)	3 (2.6)	2 (4.0)	0.230	0.632
CPB time (minutes), mean ± SD	81.09 ± 24.85	89.56 ± 12.14	3744	0.002†
XC time (minutes), mean ± SD	65.84 ± 15.87	67.64 ± 20.06	3240	0.195†
MRA time (hours), mean ± SD	10.41 ± 14.68	5.82 ± 2.44	2.195	0.030‡
ICU stay (hours), mean ± SD	55.96 ± 32.56	39.92 ± 8.62	4.886	0.001‡
Hospital Stay (days), mean ± SD	8.77±3.3	7.84±8.04	1.047	0.297‡

## Discussion

The promoted benefits of the minimally invasive approaches include smaller incision and scar sizes, less pain and trauma, less bleeding, reduced postoperative times, reduced recovery times, and increased patient satisfaction.

Working in a smaller operative field can potentially lead to prolonged operative times and may negatively impact the outcomes of standard aortic valve replacement. Despite being limited, randomized studies have demonstrated the benefits of minimally invasive AVR (MIAVR). Minimally invasive approaches are associated with reductions in pain, analgesic use, blood transfusion, mechanical ventilation time, hospital stays, financial costs, and positive cosmetic outcomes. These benefits occur without compromising the safety and quality of the AVR procedure [6-11].

In a meta-analysis by Khalid et al., 48 studies were included with a total of 17, 269 patients [12]. In comparison to standard sternotomy, there was no statistically signiﬁcant difference in in-hospital mortality in ministernotomy (RR:0.80; 95% CI:0.50-1.27; I 2 = 1%; P = 0.42) and right mini-thoracotomy (RMT) (RR:0.70; 95% CI:0.36-1.35; I^2^ = 0%; P = 0.29). Conventional sternotomy was also linked with a significantly longer cardiopulmonary bypass duration than ministernotomy (mean difference: 8.68; 95% CI:5.81-11.56; I^2^ = 92%; P = 0.00001). They found that hospital length of stay was found to be shorter in ministernotomy. The most extensive studies are retrospective, propensity score-matching, and meta-analytic. In another large meta-analysis of 42 studies with 14,925 patients, Ogami et al. found that the operative mortality was significantly lower in ministernotomy compared to conventional sternotomy [13]. The hospital length of stay was significantly shorter in the MS group compared to the CS [13]. A very large propensity score match study presented in 2015 by Neely et al. confirmed most of the clinical benefits of MIAVR via ministernotomy, which include decreased transfusion requirements, ventilation times, and ICU and hospital stays, all without compromising short- and long-term survival compared with conventional AVR via full sternotomy [14]. They conducted a propensity-score matched analysis since 2002, resulting in 552 matched pairs. The median cardiopulmonary bypass and cross-clamp times were shorter in the ministernotomy group: 106 minutes vs. 124 minutes (P ≤ 0.001) and 76 minutes vs. 80 minutes (P ≤ 0.005), respectively. Ministernotomy patients experienced shorter ventilation times of 5.7 hours vs. 6.3 hours (P ≤0.022), shorter ICU stay times of 42 hours vs. 45 hours (P ≤ 0.039), and shorter hospital length of stay of six days vs. seven days (P ≤ 0.001) compared with the full sternotomy group. There were no differences in short- or long-term survival. These findings indicate that ministernotomy is not less effective than the standard AVR.

In a meta-analysis by Shehada et al. of nine propensity-matched score studies that enrolled 4,558 patients, 2,279 (50%) underwent standard AVR, and 2,279 (50%) underwent MIAVR [15]. They recorded a significantly lower rate of postoperative low output syndrome (1.4% vs. 2.3%, P = 0.05) and atrial fibrillation (11.7% vs. 15.9%, P = 0.01) in the MIAVR group than in the conventional group, and the minimally invasive approach increased patient satisfaction due to the smaller incision and improved cosmetic outcome. Another meta-analysis of 50 comparative studies involving approximately 12,700 patients corroborated the benefits of MIAVR [16]. The study revealed that MIAVR was associated with significantly reduced transfusion incidence (36.0% vs. 52.4%, P = 0.001) and, importantly, less postoperative pain.

There are many clinical benefits to using minimally invasive approaches, but the primary argument is the safety of this technique in terms of early mortality. The comparative mortality of minimally invasive vs. standard median sternotomy has been reported in various studies to be 5.6% vs. 7.3% (ns) [17], 2.6% vs. 4.4% (ns) [18], 1.64% vs. 1.64% [19], and 2.5% vs. 3.4% [14] or 1.5% vs. 2.2% [15], and 1.9% vs. 3.3% [16]. The early mortality rate associated with the minimally invasive approach is not higher than that of conventional surgery and, in some instances, even lower.

However, certain groups disagree with the notion that ministernotomy provides advantages. Szwere et al. reported that MIAVR does not inherently possess advantages other than a cosmetic benefit [20]. This benefit is offset by longer clamp hours, circulation time, and total surgery time [9,21]. A more profound conclusion was deduced by Nair et al. in their study conducted in the United Kingdom, where 222 patients were recruited and randomized [22]. They found that, in comparison, ministernotomy did not lead to shorter hospital stays, faster recovery, or improved survival and was not cost-effective; however, even though they were skeptical about the use of minimally invasive techniques, the primary outcomes of AVR, such as hospital mortality and major perioperative complications, were not adversely affected by the minimally invasive technique.

Several studies state comparable results between the two groups. Gilmanov et al.'s study employed a propensity-matched analysis to compare standard sternotomy and ministernotomy groups with comparable preoperative characteristics [19]. They discovered that ministernotomy had longer cardiopulmonary bypass time and cross-clamping time but shorter assisted ventilation and shorter hospital stays. The operative mortality was identical in both groups (1.64 vs1.64%; p=1.0).

Limitations

There are some limitations in this study. First, it was a single institution study and second, the sample size was small. 

## Conclusions

The minimally invasive approach is as safe as standard aortic valve surgery. Early mortality and perioperative major complications occurred without significant differences between the two groups. The duration of the cardiopulmonary bypass was longer in the ministernotomy group, but it did not influence our postoperative results. Ministernotomy is related to significantly shorter ICU stay time and ventilatory assistance, which can translate into lower hospital costs. Ministernotomy is a highly reproducible approach that can be performed using the same devices as standard surgery. We suggest incorporating minimally invasive techniques in the surgical strategies of aortic valve surgery.
